# Antimicrobial efficacy of different concentration of sodium hypochlorite on the biofilm of *Enterococcus faecalis* at different stages of development

**DOI:** 10.4317/jced.53158

**Published:** 2016-12-01

**Authors:** Mohammad Frough-Reyhani, Negin Ghasemi, Mohammadhosien Soroush-Barhaghi, Mahsa Amini, Yousefreza Gholizadeh

**Affiliations:** 1 Associate Professor, Dental Faculty, Tabriz University (Medical Sciences), Tabriz, Iran; 2 Assistant Professor, Department of Endodontics, Dental and Periodontal Research Center, Dental Faculty, Tabriz University (Medical Sciences), Tabriz, Iran; 3 Assistant Professor, Department of Microbiology, Medical Faculty, Tabriz University (Medical Sciences), Tabriz, Iran; 4 Endodontist at private practice, Tabriz, Iran; 5 Private practice, Tabriz, Iran

## Abstract

**Background:**

Persistent infection of the root canal due to the presence of resistance bacterial species, such as *Enterococcus faecalis*, has always been one of the most important reasons for endodontic treatment failure. This study investigated the antimicrobial efficacy of 1%, 2.5 % and 5% sodium hypochlorite in eliminating *E. faecalis* biofilms at different stages of development.

**Material and Methods:**

In this study 4-, 6- and 10-week-old *E. faecalis* biofilms were subjected to one of the following approaches: phosphate-buffered saline solution (PBS) or 1%, 2.5% and 5% NaOCl. Dentin chip suspensions were used for colony forming unit (CFU) counting to estimate remaining *E. faecalis* counts. Statistical comparison of the means was carried out with Kruskal-Wallis test, and pair-wise comparisons were made by Mann-Whitney U test, at a significance level of *P*<0.05.

**Results:**

The results showed that 2.5% and 5% NaOCl completely eliminated *E. faecalis* biofilms in three stages of biofilm development, whereas 1% NaOCl resulted in 85.73%, 81.88% and 78.62% decreases in bacterial counts in 4-, 6- and 10-week-old biofilms, respectively, which was significantly more than those with PBS (*p*<0.05).

**Conclusions:**

The bacteria in mature and old biofilms were more resistant to 1% NaOCl than were the bacteria in young biofilms. Overall survival rate and residual bacteria increased with biofilm aging.

** Key words:**Antibacterial, biofilm, E. faecalis, sodium hypochlorite.

## Introduction

The principal aim of root canal therapy is to eliminate bacteria from the root canal to prevent apical periodontitis or treat existing periapical disease; chemo mechanical debridement is the main technique to achieve this aim (1). Persistent infection of the root canal due to the presence of resistance bacterial species, such as *Enterococcus faecalis*, has always been one of the most important reasons for endodontic treatment failure (2). This microorganism is a gram-positive facultative anaerobic bacterial species, which is the most common species in the majority of resistant root canal infections (3). Some of the prominent features of this bacterial species are resistance to antibacterial agents, resistance to acidic and basic environments, the ability to invade dentinal tubules and adhere to dentin surfaces, and the ability to proliferate at a wide range of temperature variations and also in the presence of highly saline solutions (3-7).

Bacteria are present in the root canal system in planktonic and biofilm forms. One of the other characteristics of *E. faecalis* is its great ability to form a biofilm (8). A biofilm is a dynamic structure of bacterial populations surrounded in a polysaccharide polymeric matrix. The resistance of bacteria in the biofilm to antimicrobial agents is 1000-1500 times that of the planktonic state (1). The cells present in the biofilm structure undergo phenotypic changes under the influence of physiologic and metabolic factors of the environment over time and create cells with more resistant phenotypes (5,6,9). Studies on the biofilm structure of *E. faecalis* have shown that after 6 weeks some signs of mineralization and complete maturation are observed in the structure of biofilm and a period of 6 weeks is considered a time interval for the maturation of biofilm (6). Sodiumhypochlorite is one of the most commonly used irrigation solutions in endodontics, which is used at a concentration of 0.5-5.25%. Its principal properties are its antibacterial activity due to its proteolytic potential and its tissue-dissolving capacity; however, its disadvantages are its toxicity and unfavorable odor and taste (10,11).

In previous studies, higher concentrations of sodium hypochlorite have been effective in eliminating *E. faecalis*. In addition, it has exhibited higher antimicrobial activity in comparison to chlorhexidine, phosphoric acid, EDTA, MTAD, tetraclean, SmearClear and polymers containing Ag/ZnO, Ag and ZnO nanoparticles (2,7,9,12-15).

It should be pointed out that the majority of previous studies have evaluated the planktonic state and immature biofilms of *E. faecalis* and mature and old biofilms have drawn less attention. The aim of the present study was to evaluate the bactericidal effects of 1%, 2.5% and 5% sodium hypochlorite on 4-, 6- and 10-week-old *E. faecalis* biofilms.

## Material and Methods

The design of this study was approved in Tabriz Dental and Periodontal Research Center’s investigation committee (No:65/2015).

-Preparation of Samples

Ninety-six human maxillary central incisors, extracted due to periodontal diseases, were selected. The roots were fully developed, mature and straight, with no root caries or prior root canal treatment. Presence of only one canal was confirmed using two radiographs (from mesiodistal and buccolingual directions). The teeth were stored in 0.5% chloramine T solution. All the attached soft tissue remnants were removed from the tooth surfaces using a periodontal curette (Hu-Friedy, Chicago, IL, USA) and the teeth were stored in 0.5% chloramines T solution until used for the purpose of the study.

The tooth crowns were removed atcemento-enamel junction (CEJ) level with a diamond disk (SP 1600 Microtome, Leica, Nu Block, Germany) and the working length (WL) was determined at 1 mm away from the apical foramen, using a #15 K-file (DentsplyMaillefer, Ballaigues, Switzerland). The root canals were preparedusing the crown-down technique, using RaCe rotary systemas follows: #40/0.10 for the coronal third, #35/0.08 for the middle third and #30/0.06 for preparation up to the WL. Each root canal was flushed with physiologic serum during instrumentation using a 2-mL syringe and a 30-gauge needle.

After instrumentation, 5.25% NaOCl solution was used for 3 minutes; then each root was immersed in 1mL of 17% EDTA for 3 minutes, followed by a final flush with phosphate-buffered saline solution (PBS).

-Microbiologic Procedures

The tooth samples were autoclaved at 121°C and 15 psi for 20 minutes to kill all the organisms. Immediately, all the teeth were transferred into brain-heart infusion broth (BHIB) (Merck, Darmstadt, Germany) at 37°C for 24 hours to make sure of the efficacy of the sterilization procedure. For the biofilm experiment, a pure culture of microorganisms was prepared in BHIB and incubated at 37°C overnight under 10% CO2. Then the bacteria were harvested by centrifugation and suspended. Cell counts were determined using a UV VISIBLE spectrophotometer at a wave length of 600 nm; one optical density unit equals 108 cells/mL (equivalent to 0.5 McFarland solution). Then, each root was placed in a sterile tube containing 2mL of standard suspension of *E. faecalis*. Every other day, fresh nutrient was added to ensure the nutritional support and stability of the media, while the temperature was preserved at 37°C. After 4, 6 and 10 weeks of biofilm growth, the root samples were randomly selected and divided into four groups (n=10), as described:

Group I: phosphate-buffered saline solution (Control)

Group II: 5% NaOCl

Group III: 2.5% NaOCl

Group IV: 1% NaOCl

In group 1, the root canals were irrigated with 10mL of PBS solution for 10 minutes. In groups 2, 3 and 4 the root canals were filled with 5.25%, 2.5% and 1% NaOCl, respectively. The solution was removed after 10 minutes, using sterile paper points, and irrigated with normal saline solution.

Subsequently, all the samples were placed in a refrigerator at -25°C overnight. The resultant precooling prevented *E. faecalis* killing because of drilling heat during the subsequent step. After 24 hours, a thin layer of internal root canal surfaces was removed using Gates-Glidden drills #5 and #6. Dentin chips were weighed with a sensitive electronic weighing machine (ASD Co., LTD, Japan) and 10mg of dentin chips were weighed for each sample. Then, the chips were placed in sterile tubes and 2mL of physiologic serum was added to each tube and mixed for 20 seconds. Ten-foldserial dilutions were made up to a concentration of 10-7. In the next stage, 10 mL of each concentration were transferred into three Mueller-Hinton agar plates. The plates were then incubated at 37°C for 48 hours. The number of colony forming units (CFUs) per mL was measured for the three plates at 10-2, 10-3 and 10-4 concentrations.

-Statistical Analysis 

Statistical comparison of the means was carried out with Kruskal-Wallis test, and pair-wise comparisons were made by Mann-Whitney U test, at a significance level of *P*<0.05.

## Results

Kruskal-Wallis test showed the significant effect of the type of antibacterial solution and biofilm age on eliminate of biofilms from the root canals (*p*=0.01 and *p*=0.03, respectively). Sodium hypochlorite at 2.5% and 5% concentrations completely eliminated 4-, 6- and 10-week-old *E. faecalis* biofilms, yielding a CFU count of zero. There were significant differences in antibacterial activities of these two solutions and 1% solution and the control group (phosphate-buffered saline solution); 1% NaOCl resulted in 85.73%,81.88% and 78.62% decreases in bacterial counts in 4-, 6- and 10-week-old biofilms, respectively, which were significantly more than those with PBS, ( Table 1).

**Table 1:**
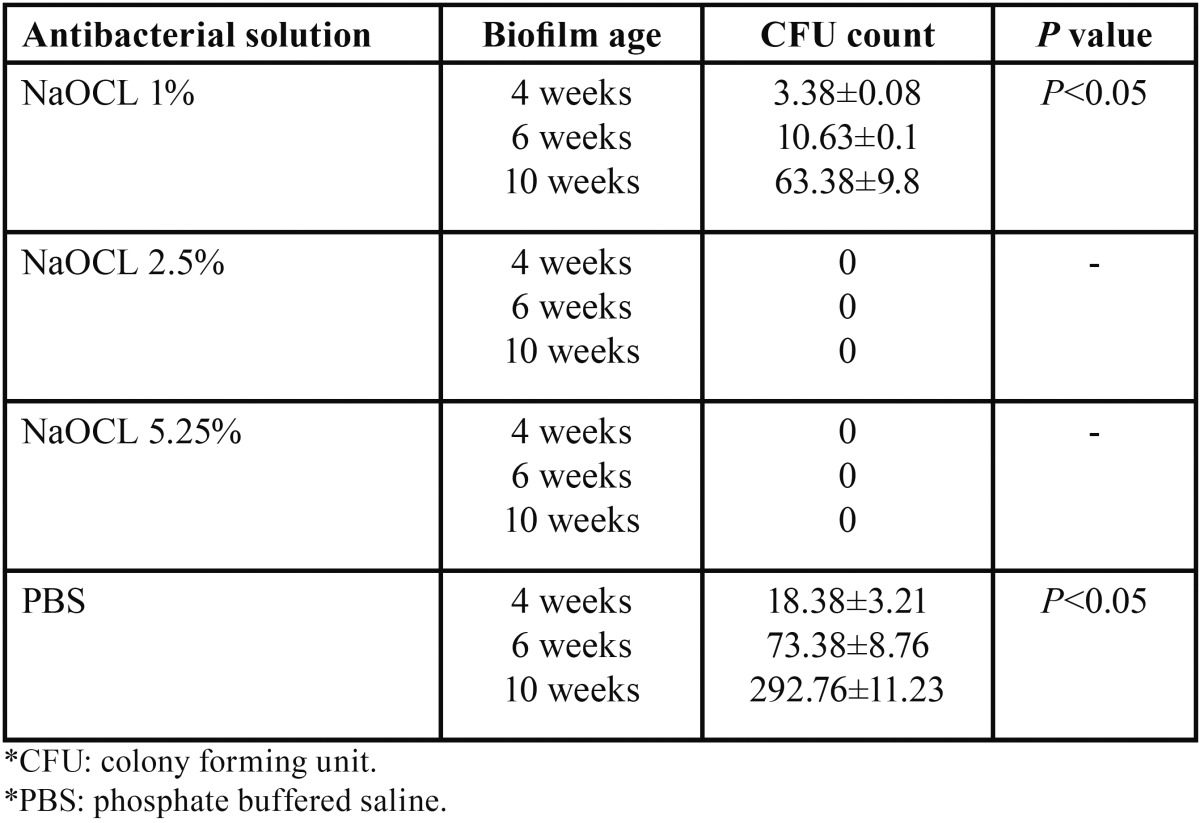
Mean±standard deviation of CFU in study groups.

## Discussion

The present study was designed with the null hypothesis that “there is no difference in the sensitivity of 4-, 6- and 10-week-old *E. faecalis* biofilms to the antibacterial effect of NaOCl at different concentrations of 1%, 2.5% and 5%”. The result of the study refuted the initial hypothesis, demonstrating that 1% NaOCl was not able to completely eliminate young, mature and old biofilms of *E. faecalis* and was only able to decrease bacterial counts compared to the phosphate-buffered saline solution.

The chief aim of root canal treatment is to eliminate bacteria, as the main etiologic factors for pulp and periapical diseases, from the root canal system (2). Bacteria have been reported in two forms of planktonic and biofilms, with the latter being a major challenge in the face of antibacterial procedures during endodontic treatment due to its high resistance against antimicrobial agents (16). On the other hand, the presence of resistant bacterial species such as *E. faecalis*, which is the predominant species in resistant and recurrent endodontic infections, increases the difficulty of root canal disinfection (1,10).

Previous studies have shown that the resistance of biofilms to antimicrobial agents increases with its aging (6,15,17,18). Therefore, in the present study biofilms were used instead of bacteria in their planktonic state. The biofilm ages, too, were defined as young, mature and old. The old age of biofilms was defined as 10 weeks of age; however, it was a maximum of 8 weeks in previous studies. *E. faecalis* is considered one of the most resistant bacterial species of the root canal system (6,14), which was used in the present study. Based on previous studies, this bacterial species has low pathogenicity in its isolated and monoclonal form; however, when it is associated with other species, it exhibits high pathogenicity. For example, its ability for commensalism with Fusobacterium nucleatum increases its resistance to harsh environmental conditions (19). In this study, one bacterial species was used, while endodontic infections have a mixed nature. This is considered one of the limitations of the present study.

The results of the present study showed that after application of 1% NaOCl solution the bacterial colony counts in the 10-week-old biofilms were higher than those in the 6-week-old biofilms, which in turn exhibited higher counts than the 4-week-old biofilms, consistent with the results of previous studies (9-10,20). These findings show that with an increase in incubation time and formation of a more mature biofilm, elimination of bacteria of an organized biofilm becomes more difficult due to the calcified and very organized structure of the older biofilm. In addition, a mature biofilm provides a special environment which isin turn a protective factor for bacteria against destructive agents such as bactericidal irrigation solutions (6). After 6 weeks of bacterial incubation, the carbonate and apatite phosphate bonds increase gradually on the biofilm surface and the biofilm exhibits a supersaturated structure (6,15). Higher concentrations used in the present study were able to completely eliminate mature and old biofilms. The efficacy of 2.5% and 5% concentrations in eliminating 6-and 8-week-old bioflms has been demonstrated in previous studies (7,10); however, no previous studies have evaluated 10-week-old biofilms.

The antibacterial effect of NaOCl solution depends on its concentration, the duration of contact with the solution, its agitation technique and how it is activated (11,13). In the present study, the contact time was 10 minutes in all the groups. At present, rotary instruments are used for debridement and shaping of root canals, which are able to prepare the root canals in less than 10 minutes (21). Therefore, it appears during instrumentation of the root canals with rotary instruments use of 1% solution is not a good choice. It is possible that the efficacy of 1% solution increases with an increase in contact time; however, further studies are necessary in this respect. In addition, it is necessary to carry out a similar study by agitating the solution (22,23). The present study was carried out on straight roots. However, based on some previous studies the penetration of irrigation solutions into the apical areas of the root canal, especially in curved root canals, is limited (24-27). Therefore, it is recommended that similar studies be carried out in curved root canals, too, to make it possible to extend the results to real clinical conditions. Furthermore, in previous studies, application of lasers, photodynamic therapy and agitation of the irrigation solution with ultrasonic devices have drawn attention as antimicrobial adjuncts and it has been reported that they increase penetration of irrigation solutions into more apical areas and root canal irregularities (20,28). It is suggested that the efficacy of these procedures in association with NaOCl, as the most effective canal irrigation solution, be evaluated in relation to old and mature biofilms and mixed bacterial species of the root canal system so that the best antimicrobial protocol can be identified.

Under the limitations of the present study it can be concluded that 1% NaOCl is not a proper choice for the complete elimination of old and mature biofilms which are predominantly found in clinical conditions, especially in resistant infections despite the fact that use of its low concentrations is always recommended due to its toxicity. On the other hand, there was no significant difference in the antimicrobial efficacy of 2.5% and 5% concentrations of NaOCl, making it justifiable to use 2.5% concentration of NaOCl.

## References

[1] Frough Reyhani M, Rahimi S, Fathi Z, Shakouie S, Salem Milani A, Soroush Barhaghi MH (2015). Evaluation of Antimicrobial Effects of Different Concentrations of Triple Antibiotic Paste on Mature Biofilm of Enterococcus faecalis. J Dent Res Dent Clin Dent Prospects.

[2] Samiei M, Ghasemi N, Divband B, Balaei E, Hosien Soroush Barhaghi M, Divband A (2015). Antibacterial efficacy of polymer containing nanoparticles in comparison with sodium hypochlorite in infected root canals. Minerva stomatol.

[3] Tagelsir A, Yassen GH, Gomez GF, Gregory RL (2015). Effect of Antimicrobials Used in Regenerative Endodontic Procedures on 3-week-old Enterococcus faecalis Biofilm. J Endod.

[4] Taneja S, Kumar P, Malhotra K, Dhillon J (2015). Antimicrobial effect of an oxazolidinone, lantibiotic and calcium hydroxide against Enterococcus faecalis biofilm: An in vitro study. Indian J Dent.

[5] Birring OJ, Viloria IL, Nunez P (2015). Anti-microbial efficacy of Allium sativum extract against Enterococcus faecalis biofilm and its penetration into the root dentin: An in vitro study. Indian J Dent.

[6] Kishen A, George S, Kumar R (2006). Enterococcus faecalis-mediated biomineralized biofilm formation on root canal dentine in vitro. J Biomed Mater Res A.

[7] Chavez de Paz LE, Bergenholtz G, Svensater G (2010). The effects of antimicrobials on endodontic biofilm bacteria. J Endod.

[8] Dunavant TR, Regan JD, Glickman GN, Solomon ES, Honeyman AL (2006). Comparative evaluation of endodontic irrigants against Enterococcus faecalis biofilms. J Endod.

[9] Stojicic S, Shen Y, Haapasalo M (2013). Effect of the source of biofilm bacteria, level of biofilm maturation, and type of disinfecting agent on the susceptibility of biofilm bacteria to antibacterial agents. J Endod.

[10] Wang Z, Shen Y, Haapasalo M (2012). Effectiveness of endodontic disinfecting solutions against young and old Enterococcus faecalis biofilms in dentin canals. J Endod.

[11] Mohammadi Z (2008). Sodium hypochlorite in endodontics: an update review. Int Dent J.

[12] Oliveira DP, Barbizam JV, Trope M, Teixeira FB (2007). In vitro antibacterial efficacy of endodontic irrigants against Enterococcus faecalis. Oral Surg Oral Med Oral Pathol Oral Radiol Endod.

[13] Rahimi S, Janani M, Lotfi M, Shahi S, Aghbali A, Vahid Pakdel M (2014). A review of antibacterial agents in endodontic treatment. IranEndod J.

[14] Siqueira JF Jr, Rocas IN, Favieri A, Lima KC (2000). Chemomechanical reduction of the bacterial population in the root canal after instrumentation and irrigation with 1%, 2.5%, and 5.25% sodium hypochlorite. J Endod.

[15] Shen Y, Stojicic S, Haapasalo M (2011). Antimicrobial efficacy of chlorhexidine against bacteria in biofilms at different stages of development. J Endod.

[16] Diogo P, Goncalves T, Palma P, Santos JM (2015). Photodynamic Antimicrobial Chemotherapy for Root Canal System Asepsis: A Narrative Literature Review. Int J Dent.

[17] Bulacio Mde L, Galván LR, Gaudioso C, Cangemi R, Erimbaue MI (2015). Enterococcus Faecalis Biofilm. Formation and Development in Vitro Observed by Scanning Electron Microscopy. Acta Odontol Latinoam.

[18] Palaniswamy U, Lakkam SR, Arya S, Aravelli S (2016). Effectiveness of N-acetyl cysteine, 2% chlorhexidine, and their combination as intracanal medicaments on Enterococcus faecalis biofilm. J Conserv Dent.

[19] Misuriya A, Bhardwaj A, Bhardwaj A, Aggrawal S, Kumar PP, Gajjarepu S (2014). A comparative antimicrobial analysis of various root canal irrigating solutions on endodontic pathogens: an in vitro study. J Contemp Dent Pract.

[20] Zand V, Milani AS, Amini M, Barhaghi MH, Lotfi M, Rikhtegaran S (2014). Antimicrobial efficacy of photodynamic therapy and sodium hypochlorite on monoculture biofilms of Enterococcus faecalis at different stages of development. Photomed Laser Surg.

[21] Lotfi M, Vosoughhosseini S, Saghiri MA, Zand V, Ranjkesh B, Ghasemi N (2012). Effect of MTAD as a final rinse on removal of smear layer in ten-minute preparation time. J Endod.

[22] Llena C, Cuesta C, Forner L, Mozo S, Segura JJ (2015). The effect of passive ultrasonic activation of 2% chlorhexidine or 3% sodium hypochlorite in canal wall cleaning. J Clin Exp Dent.

[23] Niewierowski RS, Scalzilli LR, Morgental RD, Figueiredo JA, Vier-Pelisser FV, Borba MG (2015). Bovine Pulp Tissue Dissolution Ability of Irrigants Associated or Not to Ultrasonic Agitation. Braz Dent J.

[24] Akhlaghi NM, Dadresanfar B, Darmiani S, Moshari A (2014). Effect of master apical file size and taper on irrigation and cleaning of the apical third of curved canals. JDent (Tehran).

[25] Charara K, Friedman S, Sherman A, Kishen A, Malkhassian G, Khakpour M (2016). Assessment of Apical Extrusion during Root Canal Irrigation with the Novel GentleWave System in a Simulated Apical Environment. J Endod.

[26] Freire LG, Iglecias EF, Cunha RS, Dos Santos M, Gavini G (2015). Micro-Computed Tomographic Evaluation of Hard Tissue Debris Removal after Different Irrigation Methods and Its Influence on the Filling of Curved Canals. J Endod.

[27] Lorencetti KT, Silva-Sousa YT, Nascimento GE, Messias DC, Colucci V, Abi Rached-Junior F (2014). Influence of apical enlargement in cleaning of curved canals using negative pressure system. Braz Dent J.

[28] Gutknecht N, Al-Karadaghi TS, Al-Maliky MA, Conrads G, Franzen R (2016). The Bactericidal Effect of 2780 and 940 nm Laser Irradiation on Enterococcus faecalis in Bovine Root Dentin Slices of Different Thicknesses. Photomed Laser Surg.

